# Comparative Analysis of Keratometric and Pachymetry Values From Corneal Topography Scans: A Comparison Between Pentacam and Galilei

**DOI:** 10.7759/cureus.61993

**Published:** 2024-06-09

**Authors:** Usman Tariq, Shagufta Parveen, Salman Mahmood Wazir, Taimoor A Khan, Muhammad A Zahid, Muhammad Tariq Bangash

**Affiliations:** 1 Ophthalmology, Armed Forces Institute of Ophthalmology, Rawalpindi, PAK; 2 General Practice, National University of Medical Sciences, Rawalpindi, PAK; 3 Ophthalmology, National University of Medical Sciences, Rawalpindi, PAK; 4 Ophthalmology, Combined Military Hospital, Peshawar, PAK; 5 Ophthalmology, Monash Health, Clayton, AUS; 6 Ophthalmology, Islamic International Medical College, Sialkot, PAK

**Keywords:** pachymetery, keratometric, comparison, pentacam, galilei

## Abstract

Objective

The objective of this study was to compare K1, K2, Kmax, and pachymetry values from Pentacam and Galilei scans of corneal topography in order to assess their correlation and interchangeability in clinical practice.

Methodology

A total of 34 patients (68 eyes) were enrolled in the study. Corneal topography was performed using Pentacam and Galilei devices on the same day. K1, K2, Kmax, and pachymetry readings were obtained from the scans and analyzed using paired t-tests and Bland-Altman plots.

Results

There were minimal differences in clinical settings between Pentacam and Galilei for K1, K2, Kmax (>0.75 D), and pachymetry values (>15 um). However, there was a statistically significant difference found between Kmax and pachymetry, making their interchangeability questionable.

Conclusion

Pentacam and Galilei demonstrate a good correlation between corneal keratometric values (K1, K2, and Kmax) and pachymetry values in clinical settings, and they should be used interchangeably with caution.

## Introduction

The corneal shape is of paramount importance because it determines how the eye refracts the light that falls onto it and has a huge bearing on visual acuity and corneal health. Clinicians therefore commonly rely on measuring the contours of the cornea to detect several ocular disorders, ranging from refractive errors to corneal ectasias. Keratometry started with simple Placido discs, which would highlight irregularities in corneal shape in the form of irregular concentric rings. However, advancements in the field of ophthalmology have permitted us to not solely rely on anterior reflection. Modern machines use high-definition cameras to capture multiple three-dimensional (3D) pictures of the cornea and provide in situ analysis, thus revolutionizing the way we can assess the shape and health of corneas [1]. Clinicians now have access to intricate and proven tools for corneal evaluation, which enable precise keratometric assessment. The two most widely available machines are Pentacam and Galilei. The most important function of the cornea is refracting light onto the retina and thus making an image. Understanding corneal biomechanical properties is essential for diagnosing and managing keratoconus (KC), glaucoma (central corneal thickness (CCT)), and the detection and management of refractive errors. Among these, keratometry values (K1, K2, and K max) and pachymetry are the most significant in clinical practice since they are the key values that can help us diagnose KC. Both Pentacam and Galilei offer a comprehensive assessment of corneal structures, including K-values and pachymetry. Therefore, understanding the comparative efficacy of the measurement of these parameters is essential for clinicians [2].

Pentacam is a rotating Scheimpflug imaging system that captures high-resolution images of the cornea, anterior chamber (AC), and lens. It enables in-depth assessment of corneal shape and keratometric parameters. Its principle involves projecting a slit, capturing the images using an offset rotating camera, and a computer system that immediately reconstructs 3D corneal shapes and compares them with a population in the same setting. Pentacam is known for its ability to provide accurate and reproducible keratometric readings. By integrating Scheimpflug imaging with precise algorithms, Pentacam calculates K values in various desired meridians and detects corneal irregularities and astigmatism, corneal pachymetry, AC analysis, and wavefront analysis, rendering it a complete tool for anterior segment evaluation [3].

Galilei is a dual Scheimpflug and Placido disc-based corneal topographer that combines the simultaneous acquisition of Scheimpflug images and Placido disc, integrating them in real time to collect and analyze data. Thus, Galilei offers precise measurements of curvature, elevation maps, and pachymetry. This hybrid approach is thought to enhance the accuracy of Galilei, particularly in cases of irregular corneas and ectasias. The ability to analyze both corneal surfaces simultaneously and combine them with HD photos makes Galilei a valuable tool for diagnosing ectasias such as KC and especially post-refractive surgery ectasia. Galilei also offers detailed corneal aberrometry, AC analysis, and wavefront analysis [4,5].

Despite their shared purpose, Pentacam and Galilei employ different methodologies and imaging technologies, which may influence the correlation of corneal values taken from them. While comparing the two devices, there appear to be discrepancies and mixed opinions on the superiority of one system over the other. At the same time, factors like tear film and patient fixation monitoring technologies may further influence the reliability of keratometric measurements obtained from Pentacam and Galilei scans when used interchangeably [6].

The keratometric values K1 (flat value), K2 (steep value), and Kmax (the maximum keratometry value) are crucial parameters in the diagnosis and management of KC and have set limits for KC surgeries. These values therefore help in assessing the severity and progression of the KC while simultaneously guiding treatment, e.g., corneal collagen cross-linking (CXL), rigid contact lens fitting, or corneal transplantation. Similarly, in the planning and monitoring of refractive surgeries, these parameters become extremely important. Monitoring changes in K1 and K2 values and their differences helps in assessing the stability of the cornea and the effectiveness of treatment interventions. Kmax is particularly important in diagnosing (>52 D) and monitoring the progression of KC. An increase in Kmax over time may indicate progression, necessitating timely interventions (CXL) to halt or slow down ectasia and preserve vision [7].

In refractive surgery, particularly in procedures like laser in situ keratomileusis (LASIK) or photorefractive keratectomy, accurate measurement is essential for operative planning, feeding data, and finally predicting the postoperative corneal shape and refractive outcomes. Like in KC, K1 and K2 values play a critical role in preoperative evaluation and surgical planning, determining the amount and axis of astigmatism correction. Kmax is evaluated to ensure that the cornea has the required thickness and integrity to withstand surgery without compromising biomechanical stability. Following refractive surgery, the assessment of keratometry is crucial to monitor corneal stability, detect ectasia or regression, and ensure the long-term success of the refractive procedure. Any significant changes in post-op keratometry indicate the likelihood of complications (ectasia or regression), prompting further investigation and subsequent management [8].

Given the paramount importance of keratometry in both KC and refractive surgery, it is prudent to ensure the accuracy and consistency of measurements across platforms. Pentacam and Galilei are both advanced imaging technologies capable of providing accurate keratometric data. Differences in measurement algorithms, data collection and handling techniques, and device calibration may lead to subtle to marked variations in the obtained values. These differences could potentially impact clinical decision-making, especially where a machine other than one used for refractive surgery is present [9,10].

This study aims to compare keratometric and pachymetry readings obtained from Pentacam and Galilei scans to evaluate their correlation and interchangeability in clinical practice.

## Materials and methods

This prospective cross-sectional study was conducted at the Armed Forces Institute of Ophthalmology, Rawalpindi, Pakistan, from July 2023 to December 2023. The study protocol was approved by the institutional review board and ethical committee (approval number 308/ERC/AFIO), and written informed consent was obtained from all participants.

Inclusion criteria

The inclusion criteria were (1) patients aged 19-45 years, chosen to avoid age-related keratometric changes; (2) patients being screened for KC or scheduled for refractive surgeries; and (3) patients willing and able to undergo corneal topography scans using both Pentacam and Galilei devices.

Exclusion criteria

The exclusion criteria were (1) patients with a history of corneal surgery or trauma; (2) patients with corneal scarring or opacities that may affect corneal topography measurements; and (3) patients unable to undergo keratometry for any reason.

A total sample size of 34 patients (68 eyes) was calculated using OpenEpi software online, keeping the reference prevalence of KC to be 2.3% [11], a two-sided significance level (1-alpha) of 95, and a power (1-beta, % chance of detecting) of 80. Eligible participants underwent corneal topography scans using both Pentacam and Galilei devices on the same day, minimizing variations. Keratometric values (K1, K2, and Kmax) and pachymetry readings were obtained from the scans after ensuring the accuracy of the scan. The data was collected and recorded in a standardized format by a single examiner, ensuring accuracy and consistency. IBM SPSS Statistics for Windows, Version 25.0 (Released 2017; IBM Corp., Armonk, NY, USA) was used to analyze the data. Paired t-tests were employed to compare keratometric and pachymetry readings obtained from Pentacam and Galilei scans. The primary outcome measure was a comparison of keratometric values (K1, K2, and Kmax) obtained from Pentacam and Galilei scans to see the level of difference between them. A difference of >0.75 D between K1, K2, and Kmax was considered significant clinical disagreement. Secondary outcome measures were a comparison of pachymetry readings obtained from Pentacam and Galilei scans, and a difference of >15 um was considered a disagreement. Bland-Altman plots were constructed between keratometric measures K1, K2, K max, and pachymetry between both machines, and more than 95% of the confidence interval was considered significant agreement.

## Results

 The mean age of the participants was 27.53 ± 7.456 years. Out of 34 patients (68 eyes), males were 13 (n = 26), while females were 21 (n = 42). Mean keratometric values K1, K2, and Kmax from Pentacam and Galilei scans showed minimal differences. The mean K1 values for Pentacam were 42.86 ± 1.42 D, while those for Galilei were 42.91 ± 1.48 D. The mean K2 values for Pentacam were 44.36 ± 1.38 D, while those for Galilei were 44.43 ± 1.43 D. The mean Kmax values for Pentacam were 43.58 ± 1.29, while those for Galilei were 43.67 ± 1.35. Mean pachymetry readings for Pentacam were 535.81 ± 33.76 um and for Galilei were 532.59 ± 37.12 um. The pachymetry difference was 3.2206 ± 6.89764 um, and Pentacam overestimated the pachymetry values as compared to Galilei. Conversely, for K1, K2, and Kmax values, the mean differences were 0.048 ± 0.259, 0.073 ± 0.33, and 0.085 ± 0.22 diopters, respectively, where Galilei tends to produce slightly higher values compared to Pentacam. Significant agreement was found between K1, K2, Kmax, and pachymetry values more than 95% of the time (n = 68). The paired sample t-test results indicate that there is no statistically significant difference between Galilei and Pentacam measurements for K1 (t (68) = 1.530, p = 0.131) and K2 (t (68) = 1.839, p = 0.070). However, there is a statistically significant difference between Galilei and Pentacam measurements for K max (t (68) = 3.121, p = 0.003) and pachymetry (t (68) = -3.850, p < 0.001). Demographic data is shown in Table 1.

**Table 1:** Demographic data showing the values against each variable

Ser	Variable		n
1	Gender	Male	13 (26 eyes)
		Female	21 (42 eyes)
2	Mean age		27.53 years
3	Mean K1 Galilei		42.91 D
4	Mean K2 Galilei		44.43 D
5	Mean Kmax Galilei		43.67 D
6	Mean pachymetry Galilei		532.59 µm
7	Mean K1 Pentacam		42.86 D
8	Mean K2 Pentacam		44.36 D
9	Mean Kmax Pentacam		43.58 D
10	Mean pachymetry Pentacam		535.81 µm

**Figure 1:**
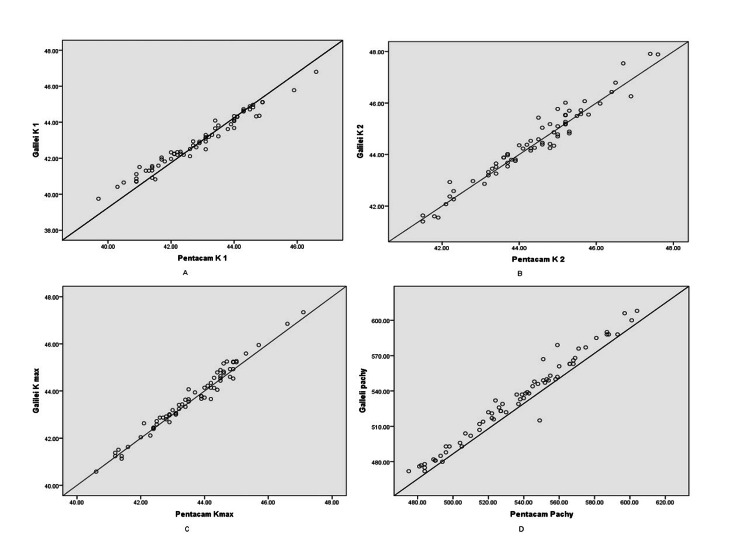
Bland-Altman plot between Galilei and Pentacam values (A) K1, (B) K2, (C) Kmax, and (D) pachymetry values lie within the 95% LOA, indicating good concordance between the measurements. LOA, limits of agreement

Bland-Altman plots shown in Figure 1A-1D were employed to examine the discrepancy between individual measurements obtained from Pentacam and Galilei devices based on K1, K2, K max, and pachymetry values. The Bland-Altman plot illustrated a notable cluster of plotted points representing differences between Pentacam and Galilei measurements falling within the 95% confidence limits for the mean difference, indicating a good agreement between the two devices, as shown above. Galilei and Pentacam exhibit substantial similarities across K1, K2, K max, and pachymetry measurements, demonstrating good agreement (within 95% limits of agreement (LOA)).

## Discussion

Corneal parameters vary with age, genetics, contact lens wear, and any medications [12]. It is imperative to measure corneal thickness and K values for accurately finding corneal power, assessment of shape and any ectatic changes, detection, monitoring, and surveillance of patients with KC and refractive surgery [13]. Our findings reveal minimal differences in keratometric values (K1, K2, Kmax, and pachymetry). However, statistically significant disparities were only measured in Kmax and pachymetry between Pentacam and Galilei. In assessing the agreement between Pentacam and Galilei G6, Bland-Altman analysis demonstrated statistically significant differences in the mean values of most parameters. However, it is crucial to note that statistical significance does not necessarily equate to clinical significance. Specifically, for corneal curvature measurement, the 95% LOA for parameters such as K1, K2, Kmax, and pachymetry. While the 95% LOA offers a range wherein 95% of differences between measurements by two methods are anticipated to fall, it is crucial to recognize that true agreement between methods extends beyond statistical analysis, and although Bland-Altman plots serve as robust tools for assessing agreement between different measurement techniques, the practical and acceptable level of agreement must ultimately be determined within the clinical context.

Multiple studies have assessed the interchangeability of Pentacam and Galilei for axial length and AC parameters. However, in clinical practice, all these parameters must be seen together rather than in isolation, especially for a patient with KC, because one single parameter is not sufficient to provide the complete picture alone. Supiyaphun et al. compared anterior segment indices, including CCT, measured by Pentacam and Galilei devices [14]. They found Galilei measurements to be slightly thicker than those of Pentacam, with a wide range of LOA, suggesting non-interchangeability between the two systems, which has also been demonstrated in multiple other studies [15].

Hsieh et al. investigated the agreement between corneal power and thickness measurements obtained from the Pentacam and Galilei systems in post-LASIK corneas [16]. They found consistency between Galilei-derived corneal power values and those obtained by Pentacam, with no significant differences in CCT. This aligns with our study’s focus on comparing corneal measurements between Pentacam and Galilei, which also demonstrated good agreement in some parameters but highlighted disparities in others.

Lanza et al. compared corneal pachymetry values measured by three different devices, Orbscan II, Pentacam HR, and Sirius, in 102 healthy eyes. The difference found between Sirius and Pentacam in CCT measurement did not appear to be statistically significant, in agreement with conclusions from other studies [17]. The CT values provided by Orbscan II were lower than those provided by Pentacam HR and Sirius, consistent with previous studies [18-21]. This discrepancy in measurements could stem from differences in measurement algorithms, acquisition techniques, and software processing between Pentacam and Galilei devices. Pentacam HR analyzes 25,000 points per acquisition, whereas Galilei can acquire over 120,000 points, potentially leading to variations in measurement precision and accuracy [22].

Despite these challenges, Scheimpflug devices offer several advantages over traditional ultrasound pachymetry, including non-contact measurement, faster acquisition times, and the ability to visualize corneal morphology in three dimensions. Additionally, Scheimpflug technology allows for the assessment of anterior segment parameters beyond CCT, such as keratometry and corneal topography, making it valuable for preoperative planning in corneal refractive surgery and the diagnosis of corneal diseases like KC [23].

Through robust statistical analysis employing paired sample t-tests and Bland-Altman plots, we have strengthened the reliability of our findings. Our study thus presents a thorough comparison of keratometric values and pachymetry readings obtained from Pentacam and Galilei devices, elucidating their concordance and disparities.

Limitations

Our study is constrained by its relatively modest sample size and single-center design, potentially limiting the generalizability of our conclusions. To address these limitations, future research is warranted to incorporate larger sample sizes, multicenter collaborations, and longitudinal follow-up to assess the predictive value of the measurements and their interchangeability over time. Moreover, exploring additional parameters and comparing them across various additional Scheimpflug-based platforms and comparing them with AS OCT would offer a more comprehensive perspective.

## Conclusions

While Pentacam and Galilei devices demonstrate good agreement for certain corneal parameters (K1 and K2), discrepancies exist, particularly in Kmax and CCT measurements. However, both devices appear to be providing clinically agreeable values and may be used interchangeably with good accuracy in clinical settings, as the difference was less than 0.75 D in keratometric values. However, these differences highlight the need for careful consideration when interpreting measurements obtained from different Scheimpflug devices and emphasize the importance of device-specific validation and calibration, especially in cases of refractive surgery where extreme precision is required in micrometers.
